# Rankings matter: nurse graduates from higher-ranked institutions have higher productivity

**DOI:** 10.1186/s12913-017-2074-x

**Published:** 2017-02-13

**Authors:** Olga Yakusheva, Marianne Weiss

**Affiliations:** 1 Department of Systems, Populations, and Leadership, School of Nursing, 400 North Ingalls Street, Suite 4243, Ann Arbor, MI 48103 USA; 2 Department of Health Policy and Management, School of Public Health, 400 North Ingalls Street, Suite 4243, Ann Arbor, MI 48103 USA; 3 Institute for Health Policy Innovation, University of Michigan, 400 North Ingalls Street, Suite 4243, Ann Arbor, MI 48103 USA; 4 Marquette University College of Nursing, 530N 16th St, Milwaukee, WI 53233 USA

**Keywords:** Nursing workforce, Productivity, Quality of education

## Abstract

**Background:**

Increasing demand for baccalaureate-prepared nurses has led to rapid growth in the number of baccalaureate-granting programs, and to concerns about educational quality and potential effects on productivity of the graduating nursing workforce. We examined the association of individual productivity of a baccalaureate-prepared nurse with the ranking of the degree-granting institution.

**Methods:**

For a sample of 691 nurses from general medical-surgical units at a large magnet urban hospital between 6/1/2011–12/31/2011, we conducted multivariate regression analysis of nurse productivity on the ranking of the degree-granting institution, adjusted for age, hospital tenure, gender, and unit-specific effects. Nurse productivity was coded as “top”/“average”/“bottom” based on a computation of individual nurse value-added to patient outcomes. Ranking of the baccalaureate-granting institution was derived from the US News and World Report Best Colleges Rankings’ categorization of the nurse’s institution as the “first tier” or the “second tier”, with diploma or associate degree as the reference category.

**Results:**

Relative to diploma or associate degree nurses, nurses who had attended first-tier universities had three-times the odds of being in the top productivity category (OR = 3.18, *p* < 0.001), while second-tier education had a non-significant association with productivity (OR = 1.73, *p* = 0.11). Being in the bottom productivity category was not associated with having a baccalaureate degree or the quality tier.

**Conclusions:**

The productivity boost from a nursing baccalaureate degree depends on the quality of the educational institution. Recognizing differences in educational outcomes, initiatives to build a baccalaureate-educated nursing workforce should be accompanied by improved access to high-quality educational institutions.

## Background

In 2010, the Institute of Medicine (IOM) issued a recommendation to increase the proportion of registered nurses (RNs) with a baccalaureate degree in nursing (BSN) to 80% by 2020 [1]. In 2014, the Magnet® Recognition Program instituted a requirement for demonstrating evidence of progress toward the IOM’s recommendation as a standard for designation [2]. Currently, more than three-quarters of employers express a strong preference for BSN-prepared nurses, and almost half of hospitals and other healthcare settings in the US are now requiring new hires to have a BSN [3]. The increased demand for BSN-prepared nurses has led to growing enrollments in BSN-granting programs, with a 4.2% growth in entry-level baccalaureate programs and a 10.4% growth in RN-to-BSN enrollment between years 2013 and 2014. A record high of 320,000 students enrolled in nursing baccalaureate programs in 2014 [4].

The surge in demand for more BSN graduates was met by increased capacity in the nearly 900 established and newly accredited BSN programs nationwide [4], with sixty new RN-BSN and RN-MSN programs projected to enter the nurse education landscape in the next several years [5]. As an alternative to traditional entry-level BSN programs, over 290 accelerated BSN programs were offering a fast track to a BSN for applicants holding a non-nursing bachelor’s degree in 2014 [4], up 15% from 2012 [6]. These different degree tracks are offered by a wide range of national and regional educational institutions, with the majority of educational institutions adopting a blended classroom-online instruction format and many programs taught completely online [4]. A relatively new but quickly expanding segment of the BSN market is community colleges whose number increased over 400% from eight in 2005 to 25 in 2010, with seven states currently allowing community colleges to confer a baccalaureate degree in nursing [7]. The multitude of degree tracks, instructional formats, and degree-granting institution types serves as affirmation of the ability of the education industry to meet the increased demand for Baccalaureate-prepared nurses; however, concerns about impact on the quality of nursing education have also been raised [8, 9].

Educational quality is determined by a number of factors including faculty resources and learning environment [10–16], and it has been linked to higher worker productivity, but not in healthcare [13–16]. What we do not know is whether the quality of the degree-granting program matters for the nurse’s ability to produce desired patient outcomes. A better understanding of the relationship between the quality of nurse education and its potential to boost productivity can inform the design of an optimal policy landscape to support the ongoing transition to a BSN-educated nursing workforce. The aim of our study was to examine whether the productivity boost from a BSN-degree depends of the quality of the degree-granting institution.

## Methods

### Conceptual framework

We conceptualize the relationship between nurse productivity and the quality of nursing education using the economic theory of human capital [17], nursing intellectual capital theory [18–20], and a theory of job performance and productivity from the field of organizational psychology [21, 22]. Human capital embodies knowledge, skill, and experience attributes of an individual [17, 20]; it is viewed as a foundational antecedent of individual performance, or the capacity of an individual to carry out and accomplish job-related processes or functions [21, 22]. Individual performance, in turn, is a key determinant of individual productivity, or the contribution of an individual to the total economic production of an outcome [17, 21, 22]. Applying these concepts to nurses, a nurse’s individual performance in processes of care gives rise to individual productivity, the unique contribution of the nurse to outcomes of patients under the nurse’s direct care [18, 23]. Completion of a baccalaureate program increases the nurse’s human capital, thereby raising the nurse’s performance (process) and, consequently, productivity (outcome). We conjecture that the quality of the BSN-granting institution is a moderating influence in the relationship between nurse human capital and nurse productivity.

### Nurse contribution to outcomes

We adopt Irvine et al.’s (1998) conceptualization of the contribution of nurses to patient outcomes [24, 25]. Irvine and colleagues argue that specific patient outcomes can be attributed to nurses’ independent, dependent, and interdependent roles and functions as they collaborate with other clinicians in joint care delivery efforts. Specifically, nurses’ independent role functions have a direct impact on clinical outcomes (physiological outcomes and symptom control), functional outcomes (emotional status, cognitive status, mobility), and knowledge of self-care. However, in their dependent and interdependent role functions, nurses can ultimately affect all categories of patient outcomes. For example, while not solely accountable, nurses have a major responsibility in preventing complications (injury or falls, nosocomial infections, pressure ulcers, etc.), because they provide the majority of round-the-clock direct patient care and supervision in acute care [24, 25].

Building on this model of nursing roles and functions in collaborative care delivery efforts, this manuscript views productivity of an individual nurse as the combined contribution of the nurse’s performance in independent, dependent, and interdependent functional roles to the outcomes of patients assigned to the nurse’s direct care. The methods used recognize that patient outcome is the result of the contributions of many nurses to a single patient’s care. Through linked patient nurse records, the unique productivity contribution of each nurse to total production of patient outcomes can be identified.

### Design

The study was a retrospective secondary data analysis of the moderating effect of the quality of the BSN-granting institution on the association of type of nurse education (BSN or higher, not BSN) with a patient-outcome based measure of performance. We hypothesized that having a BSN increases the odds of high productivity, and that the positive effect would be greater if the BSN was from a higher-ranking institution (Hypothesis 1). Similarly, we hypothesized that having a BSN reduces the odds of low productivity, and that this effect would also be greater if the BSN was from a higher-ranking institution (Hypothesis 2). Nurse education (highest completed degree), the name of the degree-granting institution (for the highest completed degree), and other nurse characteristics (age, gender, experience at the hospital) were obtained from human resource (HR) data. Educational institution rankings were obtained from the U.S. News and World Report. Nurse productivity ratings were derived from computation of the nurses’ individual value-added to patient outcomes [26].

### Sample

We used de-identified data for 1,203 nurses on general medical or surgical units at an urban teaching hospital during 7/1/2011–12/31/2011. In our prior study, we had linked these nurses to over 7,300 adult medical-surgical in-patients and derived an individual nurse productivity measure for each nurse based on the outcomes of the patients linked to the nurse [26]. The current study required that we rank the productivity of each nurse relative to the other nurses on the same unit, therefore we excluded 367 nurses who worked on more than one unit during the study period; we also excluded 145 nurses with missing data on the education level. Our final sample had 691 nurses, resulting in 86–92% statistical power for large effect sizes and 69–73% power for medium effect sizes, at the conventional level of significance *p* = 0.05.

### Measures

#### Nurse productivity

Our main outcome measure was categorized as top, average, and bottom productivity. In our prior study [26], we estimated nurse productivity as the nurse’s individual value-added contribution to improvement in the patient’s clinical condition score [27–29] among patients assigned to the nurse’s care. We used Value-Added Methodology [30] to, first, attribute a change in each patient’s clinical condition score (from admission to discharge) equally to all of the nurses assigned to the patient’s care during hospitalization, and then, for each nurse, compute a risk-adjusted aggregate change in clinical condition scores of the patients linked to the nurse during the study period [26]. The clinical condition score is a composite metric of 26 clinical parameters from the patient’s electronic medical record including nurse assessments (nutritional status, skin, functional status, psycho-emotional status, pain, etc.), vital signs (temperature, blood pressure, etc.) heart rhythms, and lab tests [27–29]. In prior work, the score was shown to have construct validity for overall patient clinical condition and was predictive of discharge disposition [27], mortality [27–29], cardiac and pulmonary arrest [31], and readmission [32]. Nurse assessments, an independent functional role of a clinical nurse [24, 25], account for nearly 70% of the variance in the clinical condition score [28], supporting the use of this outcome for individual nurse productivity measurement.

Based on this previously derived individual productivity measure, nurses in the bottom and top tertile of the individual productivity distribution were categorized as “bottom” and “top” productivity categories, respectively. The middle one-third of the productivity distribution served as the reference category, or “average” productivity. The three productivity categories were associated with clinically significant differences in patient outcomes among the sample tertiles – average improvement in the patient clinical condition score was 0.03/0.48/0.91 standard deviations in the bottom/average/top productivity category, respectively; unplanned 30-day readmission rates were 18.8/16.8/15.9%; inpatient mortality rates were 4.2/2.6/2.2%.

#### Quality of BSN education

The nurses were categorized as BSN-prepared if their highest degree was a Baccalaureate degree or higher. There were no non-nursing Baccalaureate degrees in our sample. Our main exposure variable, quality tier of the degree-granting institution, was derived from the *U.S. News and World Best Colleges Report* 2014 Edition [33]. We used the 2014 Edition of *U.S. News and World Report* because this was the earliest year when significant changes to the ranking methodology were implemented that reduced the weight of factors reflecting a school’s student body and increased the weight of measures that are outcomes-based and signal the quality of education [33]. *The US News and World Report* ranks nationally accredited higher-education institutions in the U.S. and categorizes them as “first tier” (the top three-quartiles) or “second tier” (the bottom quartile); we used this two-tier categorization in our analyses [33].

*US News and World Report* collects information from over 1,500 regionally accredited U.S. institutions and ranks them using a three-step process. First, the institutions are categorized into four types of higher education institutions as defined by the Carnegie Classification [34]: National Universities, National Liberal Arts Colleges, Regional Universities, and Regional Liberal Arts Colleges. Second, the data are collected from each institution on 16 indicators of academic excellence including assessment by administrators at peer institutions, retention of students, faculty resources, student selectivity based on standardized academic assessment scores, financial resources, alumni giving, graduation rate performance and, for National Universities and National Liberal Arts Colleges only, high school counselor ratings of colleges. The indicators are assigned relative importance weights and the composite weighted scores are computed for each institution. Third, the colleges and universities in each category are ranked against their peers based on the composite score. The *US News and World Report* lists rankings for the top 75% of nationally or regionally accredited institutions within each Carnegie Classification category and classifies them as “first-tier”; the bottom 25% within each category are considered “second-tier” and their rankings are listed as “not published” [33, 35, 36]. A number of accredited institutions (less than 10%) are not ranked, either because they did not submit the required information or because they do not use standardized academic assessment scores as part of the admission process.

Fifty-three BSN-granting institutions in our sample had a published ranking as a first-tier educational institution, including 24 regionally-accredited and 29 nationally-accredited colleges and universities. Fifteen U.S. institutions in our sample were listed as second-tier. A small number nurses (*n* = 37, 5% of the sample) in our sample graduated from a non-ranked accredited or a non-accredited institution and were assigned to the second-tier category. Nurses who did not have a baccalaureate degree (i.e. were diploma or associate degree nurses) served as the reference category.

#### Control variables

We included controls for age (continuous variable measured in years), experience (continuous variables measured as number of years of tenure at the hospital), gender (1 if female, 0 if male), and fixed effects for the nursing unit to adjust for unit-level differences.

### Analysis

We calculated descriptive statistics of the sample using counts and percentages for categorical variables (gender, BSN, BSN quality tier, nurse productivity category) and means and standard deviations for continuous variables (age, experience) (Table 1).

**Table 1:** Descriptive Statistics of the sample, *n* = 691

Variable	N (%)/Mean (SD)
Sex:	
Male	73 (11)
Female	618 (89)
Age, years	33.66 (10)
Experience, years	4.36 (6.06)
Education:	
Diploma/Associate Degree	226 (33)
BSN:	465 (67)
First-tier BSN	256 (37)
Second-tier BSN	209 (30)
Productivity Category:﻿	
Top	232 (34)
Average	231 (33)
Bottom	228 (33)

Notes: For continuous variables (age and experience), we display the mean and the standard deviation

We examined the moderating effect of the quality of the educational institution on the relationship between having a BSN and the odds of being in the top and bottom productivity categories (relative to the reference category of average productivity) with a multinomial ordered logistic regression model of the nurse productivity category on the BSN quality tier, relative to not having a BSN degree. We adjusted the model for nurse characteristics (age, experience, gender) and controlled for unit-level fixed effects. Multinomial logistic regression allows for simultaneous estimation of the odds of being in the top and in the bottom productivity category, allowing for the separate analyses of predictors of high and low productivity.

Hypothesis H1:

$$ \begin{array}{l} \log \left(\frac{P\left( Top\  Productivity\ \right)}{P\left( Average\  Productivity\ \right)}\right)\\ {}={a}_0\kern0.5em +\kern0.5em {a}_1`` First\  tier\  BSN"+\kern0.75em {a}_2`` Second\  tier\  BSN"+\kern0.5em {a}_3 Age\\ {}+\kern0.5em {a}_4 Experience+\kern0.5em {a}_5 Gender\kern0.5em +\kern0.5em  Unit\  FEs\end{array} $$

Hypothesis H2:

$$ \begin{array}{l} \log \left(\frac{P\left( Bottom\  Productivity\ \right)}{P\left( Average\  Productivity\ \right)}\right)\\ {} = {b}_0+{b}_1`` First\  tier\  BSN"+\kern0.75em {b}_2`` Second\  tier\  BSN"+\kern0.5em {b}_3 Age\\ {}+\kern0.5em {b}_4 Experience+\kern0.5em {b}_5 Gender\kern0.5em +\kern0.5em  Unit\  FEs\end{array} $$

**Fig. 1:**
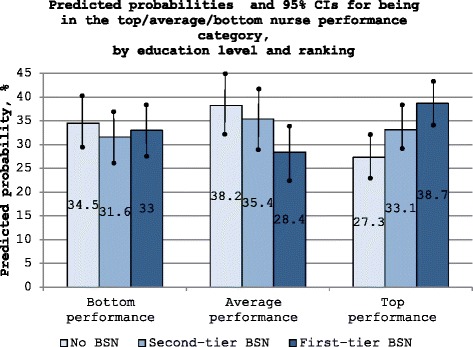
Predicted probabilities and 95% CIs (superimposed confidence intervals) for being in the top/average/bottom nurse productivity category, by education level and ranking. The figure displays the predicted probabilities of being in the top, average, and bottom productivity category (differentially shaded bars), and the corresponding 95% confidence intervals (superimposed vertical solid black lines), by the education level and tier of the registered nurse

The moderating effect of the BSN quality tier on the odds of top productivity (hypothesis 1) would be supported if the odds ratios (OR) corresponding to the coefficient of the first-tier BSN *(a*_*1*_) and to the coefficient of the second-tier BSN *(a*_*2*_) were both greater than one and the OR corresponding to the first-tier BSN was greater than the OR for the second-tier BSN. We conducted a standard *t*-test for each individual OR and a Chi-square test for comparing ORs, using two-tailed critical values for *p* < .05. Hypothesis 2 was tested similarly. We reported the ORs (Table 2) and displayed the predicted conditional probabilities of being in each of the three productivity categories (top, average, and bottom), and the corresponding 95% confidence intervals (95% CI) (Fig. 1). All analyses were conducted in Stata 14.0 [37].

**Table 2:** Odds of being in the top and in the bottom nurse productivity categories relative to the average productivity category

Characteristic	Top productivity category OR [95% CI] (*P*)	Bottom productivity category OR [95% CI] (*P*)
Education:		
No BSN	REF	REF
First-tier BSN	3.18 [1.59–6.33] (<0.001)^**^	1.19 [0.66–2.14] (0.57)
Second-tier BSN	1.73 [0.88–3.39] (0.11)	0.89 [0.49–1.63] (0.71)
Gender:		
Female	REF	REF
Male	1.00 [0.47–2.12] (1.00)	0.78 [0.37–1.67] (0.52)
Age	1.03 [1.00–1.07] (0.08)	1.00 [0.98–1.04] (0.75)
Experience	0.99 [0.94–1.04] (0.68)	0.97 [0.92–1.02] (0.24)

Notes: Estimates using an ordered logistic regression model

adjusted for unit fixed effects. ** < 0.01

## Results

Most of the nurses were female (89%), with the average age of 33.6 years and average experience at the study hospital of 4.4 years (Table 1). Two-thirds of the nurses (*n* = 465) had a BSN degree or higher, among whom more than half (*n* = 256) received the degree from a first-tier institution and the rest (*n* = 209) graduated from a second-tier institution; one third of the sample (*n* = 226) did not hold a BSN degree. A total of 232 nurses in the study sample were in the top productivity category, 231 were in the average productivity category, and 228 were in the bottom productivity category.

The analysis of the moderating effect of educational quality partially supported our hypothesis 1 that having a BSN would increase the odds of being in the top productivity category, with a greater effect for graduates from top-tier educational institutions (Table 2). Relative to nurses who did not have a BSN, BSN-prepared nurses who graduated from a second-tier institution were not more likely to be in the top productivity category (OR = 1.73; *p* = 0.11; 95% CI: 0.88–3.39), while BSN graduates from a first-tier institution had more than three-times the odds of being in the top productivity category than non-BSN graduates (OR = 3.18; *p* < 0.001; 95% CI: 1.59–6.33). The difference between the odds ratios of the first-tier from second-tier BSN graduates was significant (*x* ^2^ = 3.80, *p* = 0.05). The analysis did not support hypothesis 2 that having a BSN would decrease the odds of being the bottom productivity category. The odds ratios of being in the bottom productivity category were non-significant for both second-tier BSNs (OR = 0.89; *p* = 0.71; 95% CI: 0.49–1.63) and for first-tier BSNs (OR = 1.19; *p* = 0.57; 95% CI: 0.66–2.14) (Table 2); the difference between the ORs was non-significant (*x* ^2^ = 0.92; *p* = 0.34).

We displayed the predicted absolute probabilities and 95% CIs of being in the bottom, average, and top productivity categories by the nurse’s education level and quality tier in Fig. 1. In the absence of an association of BSN education with productivity, one would expect one-third of the nurses to be in the bottom, average, and top productivity categories, reflecting the original construction of the productivity categories. However, the probability of being a top performer was the highest among first-tier BSNs (38.7%; 95% CI: 34.5%–43.1%), with a 5.6 (*p* = 0.08) percentage point lower probability among second-tier BSN, (33.1%; 95% CI: 28.6%–37.5%), and a 11.4 (*p* < 0.01) percentage point lower probability among non-BSN nurses (27.3%; 95% CI: 22.8%–31.8%). The probability of being an average performer showed the opposite pattern with top-tier BSNs having the lowest probability (28.4%; 95% CI: 23.4%–33.2%), second-tier BSNs at 35.4% (95% CI: 29.6%–41.1%), and non-BSNs at 38.2% (95% CI: 32.3%–44.1%). The probability of being in the low productivity category had no association with educational level or quality tier, with relatively equal probabilities for non-BSNs (34.5%; 95% CI: 29.5%–39.5%), second-tier BSNs (31.6%; 95% CI: 26.7%–36.5%), and first-tier BSNs (33.0%; 95% CI: 28.4%–37.5%).

## Discussion

Our study was the first to examine the role of the quality of nursing education by linking the ranking of a nurse’s degree-granting institution to the nurse’s productivity. Our results demonstrated a quantifiable advantage of first-tier institutions over second-tier institutions in graduating top-productivity nurses. Top-productivity nurses were those with a greater overall improvement in their patients’ clinical condition, a metric associated in prior studies with lower rates of in-hospital mortality and 30-day readmission [27, 29, 32]. Linking the ranking of the BSN-granting institution to higher patient outcome-based nurse productivity speaks to the societal value of baccalaureate nurse education from a high-quality educational institution.

Our findings extend the existing evidence of significant gains in terms of patient outcomes from increasing the proportion of BSN-prepared hospital nurses [26, 38–42]. However, we also present a cautionary note that all BSN programs may not produce similar results in terms of productivity of the graduating workforce. The advantage of high-ranking institutions is likely driven by an interaction between the quality of education and the personal human capital attributes of nurses accepted to top-tier BSN programs. While further research will be useful in clarifying this complex interaction, it is imperative that access to quality education be advocated and the quality of nursing education is not sacrificed as the volume and breadth of the nursing education landscape grows to meet the increased demand for highly-educated nurses.

Being in the bottom productivity category was not associated with quality of the educational institution attended by the nurse. Baccalaureate education did not safeguard against below-average nurse productivity, with BSN nurses equally likely to be in the low productivity category relative to non-BSN level nurses (Associate Degree and Diploma) in our sample. Below-average productivity is likely attributable to other factors including poor orientation and preceptoring, job satisfaction, or personal stressors [43–46]. Further studies are required to explore the mechanisms underlying the gap in patient outcomes between top, average, and low-productivity nurses.

Our study had several limitations. First, the study was powered for large effect sizes; the finding related to the impact of second-tier BSN compared to non-BSN on productivity might be significant in a larger sample. Non-significant independent associations of age and experience with productivity may also be the results of suppression effects between these two positively correlated variables in this sample. Second, total years of experience was not recorded in the data; using years of hospital tenure as a proxy may have further contributed to non-significant findings for experience. Third, quality of education was based on the ranking of the educational institution and not the program itself; several educational institutions in our sample were not ranked and assigned to second tier; the rankings were obtained from *US News and World Report* for 2014 and might have been different at the time the nurse’s degree was conferred; the measure is subject to limitations of the *US News and World Report’s* rankings [33]. Fourth, nurse productivity might depend on unmeasured nurse-level factors (expertise, stress, etc.); we report associations that should not be interpreted as causal relationships. Fifth, the analysis was conducted at a single large Magnet®-designated teaching facility with a high proportion of BSN-prepared nurses. The analytic approach to calculating nurse productivity was developed in our prior study and has not been validated in different nurse and patient samples. Sixth, we did not examine interaction among nurses with different quality educational backgrounds; examining whether nurses with degrees from high-ranking institutions improve outcomes of other nurses was outside of the scope of this study. Further studies are required to address these limitations.

## Conclusions

In conclusion, we argue that Baccalaureate education in nursing contributes a greater boost to nurse productivity and patient outcomes when the degree is obtained from a higher-ranking educational institution. Transitioning toward the 80% BSN workforce target should focus on state and federal tuition assistance programs to improve access to high-quality regional and national educational institutions, to avoid potential dilution of quality and value of baccalaureate education through proliferation of lower quality programs. Below-average productivity was not associated with education level or quality ranking, suggesting that further research is needed to explore the antecedents of low productivity and approaches to mitigating them, in order to assure quality patient outcomes.

## Abbreviations

BSN: Baccalaureate of Science in Nursing
CI: Confidence interval
OR: Odds ratio
RN: Registered nurse
